# Quality of life questionnaire for women with gestational diabetes mellitus (GDMQ-36): development and psychometric properties

**DOI:** 10.1186/s12884-019-2614-y

**Published:** 2019-11-29

**Authors:** S. Mokhlesi, M. Simbar, F. Ramezani Tehrani, N. Kariman, H. Alavi Majd

**Affiliations:** 1 Department of medical science,Qom branch, Islamic azad University, Qom, Iran; 2 Professor, Midwifery and Reproductive Health Research Center, Shahid Beheshti University of Medical Sciences, Tehran, Iran; 3 Professor, Gynecology Department, Reproductive Endocrinology Research Center, Research Institute for Endocrine Sciences, Shahid Beheshti University of Medical Sciences, Tehran, Iran; 4 Midwifery and Reproductive Health Department, school of Nursing and Midwifery, Shahid Beheshti University of Medical Sciences, Tehran, Iran; 5 Professor, Department of Biostatistics,Faculty of Paramedical Sciences, Shahid Beheshti Medical University, Tehran, Iran

**Keywords:** Quality of life, Gestational diabetes mellitus, Questionnaire development, Psychometric properties of questionnaire

## Abstract

**Background:**

Gestational diabetes mellitus carries serious risks to mother and fetus and causes social, mental, and psychological consequences which can affect mothers’ quality of life. Accordingly, this study aims to develop and assess the psychometric properties of quality of life questionnaire for women with gestational diabetes mellitus.

**Methods:**

A methodological study of sequential exploratory mixed method was developed and implemented. It included qualitative (development of a quality of life questionnaire for mothers with GDM) and quantitative (assessment of psychometric prosperities of quality of life questionnaire for mothers with GDM) phases.

**Results:**

Based on the findings of the qualitative phase and literature review, the primary questionnaire was prepared with 142 items. The outcome of face validity and content validity assessment was a 67-item questionnaire. S-CVI and S-CVR turned out to be 0.92 and 0.68, respectively. The results of exploratory factor analysis yielded an instrument with 36 items in five domains including concerns about high-risk pregnancy, perceived constraints, disease complications, medication and treatment, and support. Five factors explained 46.68% of the total variance of the questionnaire. The results indicated a moderate and significant correlation between the questionnaire of “Diabetes Clients Quality Of Life” and the researcher-made questionnaire (r = 0.63). Cronbach’s alpha coefficient for the entire scale was 0.93 and the intra-class correlation coefficient was 0.95.

**Conclusion:**

Quality of life questionnaire for mothers with GDM is a valid and reliable tool capable of measuring the quality of life of women with GDM.

## Background

Diabetes is the most common and prevalent medical condition in pregnancy, which is defined as varying degrees of carbohydrate intolerance diagnosed or initiated during the pregnancy for the first time. This definition is applied regardless of insulin administration for treatment [1]. The prevalence of gestational diabetes is increasing worldwide including Iran; in one meta-analysis, the prevalence of Gestational Diabetes in Iran was estimated as 4.9% [2].

GDM is associated with many psychological and social consequences in addition to increased risks for mother and fetus [2, 3]. Studies have shown that after a positive screening, anxiety and worry increased in these women about the likelihood of chronic diabetes and their babies’ health (the effect of insulin or diet on the fetus), thus reducing their perception of health. Further, for some women, GDM carries a stigma and they are ashamed to express it [4–6]. A study in China indicated that approximately 260,000 QALYs (quality adjusted life years) were lost over a 3-month period due to maternal complications associated with GDM during pregnancy and childbirth [7]. All of these consequences can affect the quality of life of mothers with GDM.

Health-related quality of life represents a measure of physical and social activity as well as mental health and is considered as an important health indicator [8]. Today, the evaluation and record of health-centered quality of life via quality of life questionnaires have become crucial in medical and nursing interventions [9]. The quality of life of mothers with GDM has also been evaluated in related studies. In the study by Kepk et al., for mothers with GDM, SF-8 quality of life questionnaire was completed on weeks 27 and 36, where GDM had no significant effect on their physical, mental, occupational, or schooling dimensions [10]. Tratnowski et al. used WHOQOL-Bref questionnaire to assess the quality of life of mothers with GDM. The mean score of physical, mental, and social aspects in this questionnaire decreased significantly from mid to late pregnancy [11]. In the study by Dalfera et al., evaluating the quality of life of pregnant mothers with diabetes, the scores for mental aspect obtained from SF-36 questionnaire were not significantly different between mothers with GDM and the control, though the mothers’ physical aspect was improved [12]. One of the reasons for the contradictory results of studies on the quality of life of mothers with GDM can be that their quality of life has been measured by a general questionnaire in these studies. Although general questionnaires are valid, they may not be suitable for measuring the quality of life of a specific community such as pregnant women or women with GDM. These questionnaires may not be sufficiently sensitive to reflect the impact of small but important interventions or treatments, and may ignore the unique viewpoints of pregnant women with or without pregnancy complications. Note that during this period, specific psychological pregnancy-related changes develop along with new concerns due to the presence of the fetus [13].

A specific quality of life questionnaire is used to examine the elements relevant to a specific disease. A specific questionnaire on the quality of life of pregnant women [14, 15] as well as specific quality of life questionnaire for people with diabetes [16–18] has been developed in different countries. However, no specific quality of life questionnaire has been developed for women with GDM. The specific quality of life instrument is developed for a disease, and since these instruments are sensitive in examining small but important changes in treatments and care, they can be used to examine such effects and treatments [19]. Therefore, the quality of life questionnaire for patients with GDM can provide valuable information about the factors with the greatest impact on a person and can help find the best option in the patient care (treatment and screening). Also, the members of the healthcare team can use the quality of life index to improve interaction with patients, identify the aspects of life representing the patients’ major concerns, as well as developing and evaluating the measures taken based on the information obtained.

According to literature review on the quality of life of pregnant mothers with GDM, a specific study has not been performed for development and psychometric evaluation of quality of life questionnaire for women with GDM in Iran or in another country. Therefore, due to the high prevalence and growth of gestational diabetes in Iran and the importance of quality of life promotion in patients with GDM, this study aims to develop and assess the psychometric properties of quality of life questionnaire for women with GDM.

## Methods

A methodological study of sequential exploratory mixed method was developed and implemented. It included qualitative (development of a quality of life questionnaire for mothers with GDM) and quantitative (assessment of psychometric prosperities of quality of life questionnaire for mothers with GDM) phases. The research setting included the healthcare centers affiliated with Shahid Beheshti University of Medical Sciences in Tehran. The study sample consisted of the pregnant women with GDM. The inclusion criteria were GDM in the current pregnancy and having no other physical and psychological disorders according to patient’s statement. The sampling lasted from March 2017 to October 2018.

### Instrument development

In this study, Waltz’s 4 steps of instrument development (2010) were used for instrument development [20]. Also, the deductive-inductive method was used to extract items of the quality of life questionnaires for women with GDM (GDMQ-36). In order to explain the concept and dimensions of mothers’ quality of life with GDM, qualitative study was performed using conventional content analysis method. For this purpose, 25 pregnant women with GDM and 8 experts were individually interviewed using an unstructured questionnaire. Experts included Obstetrician, endocrinologist, dietitian, reproductive health specialist, and midwife with at least two years of experience with GDM women.

In this study, 75 g OGTT were used to screening and diagnosing gestational diabetes in the 24–32 weeks of pregnancy. Gestational diabetes was being given, if one of the numbers was equal or higher than normal limit (≥92 for fasting, ≥180 and ≥ 153 mg/dL for 1-h and 2-h plasma glucose level, resp) [21]. This information were recorded at prenatal care.

The data was analyzed by Graneheim and Lundman’s conventional content analysis method [22]. At the end of the qualitative phase of the study, the concept and dimensions of the quality of life of mothers with gestational diabetes were explained. In order to test the accuracy of the data, Lincoln and Guba’s four criteria were used including credibility, reliability, authenticity, and transferability [23].

Subsequently, extensive literature review was conducted using the keywords “Quality of Life OR Quality of life questionnaire” AND “Gestational diabetes OR Diabetes” in Scopus, Pubmed, Science Direct, Google Scholar, SID, and Magiran databases (Table 1). The inclusion criteria were Persian or English sources, inclusion of keywords in the study, and the publication of studies in prestigious domestic or foreign journals. Accordingly, the articles published in the last thirty years were reviewed and by comparing of our extracted items and those in literature and omitting the duplicated items, the most relevant neglected items were selected by expert panel to be added to the questionnaire to improve its comprehensiveness. In this way, the initial questionnaire for assessing the psychometric properties was prepared.

**Table 1:** Tools were reviewed to extract the item of quality of life questionnaire for mothers with gestational diabetes mellitus

Title of the tool	Author-year	Dimensions of the questionnaire	Face validity	Content validity	Factor analysis	Convergence Validity	internal consistency	stability
36 item short form survey (SF-36) [24]	Ware and Sherbourne- 1992	PF,^1^ RP,^2^ BP,^3^ GH,^4^ VT,^5^ SF,^6^ RE,^7^ MH,^8^	–	–	–	–	–	–
World Health Organization Quality Of Life Bref (WHO-QOL Bref) [25]	World health organization-1992	Physical health, psychological, social relationship, environment	–	–	–	–	–	–
Audit of Diabetes Dependent QoL (ADDQOL) [16]	Bradley-1999	13 item	–	–	3 factor	–	–	–
diabetes quality of life brief clinical inventory [26]	Burroughs-2004	Self care behaviors, satisfaction with diabetes control	–	–	–	–	0.77	0.72
diabetes-specific quality-of-life (D-QOL) scale [17]	Lee-2012	Emotional sufferinge, Social functioninge, Adherence to the treatment regimene, Diabetes-specific symptoms	–	Qualitative validity	4 factor with 70% variance	moderate and significant relationship between the D-QOL and SF-36	0.92%	–
Diabetic Clients Quality Of Life (DCQOL) [18]	Darvishpoor-2005	Physical, psycological, Social, Economic, Disease and treatment	–	–	5 factor with 52% variance	significant relationship between the DCQOL and SF-36	0.88–0.93	0.86–0.90
Personal Diabetes Questionnaire [27]	Stetson-2011	Diet knowledge and skills Dietary decision making Eating problems Diet barriers Problems in medication used Medication barriers Monitoring barriers Exercise barriers	–	–	–	–	–	0.65–0.83
Diabetes Management Self –Efficacy Scale [28]	Bijl-1999	physical exercise, blood sugar, nutrition general and medical treatment, nutrition specific and weight	–	–	4 factor with 55% variance	–	0.81	0.79
Diabetes Knowledge Questionnaire [29]	Garcia-2001	24 Item	–	–	–	–	0.73–0.84	–
Adherence in Diabetes Questionnaire [30]	Kristensen-2012	19 Item	–	–	1 factor	Predictive validity with HbA1C	0.82–0.86	–
Psychological Predictors of Therapeutic success in Diabetes (PPTD) questionnaire [31]	Rotella-2014	19 Item	–	–	–	Predictive validity with HbA1C	0.63	–

^1^Physical functioning

^2^role limitations due to physical problems

^3^bodily pain

^4^general health perceptions

^5^vitality

^6^social functioning

^7^role limitations due to emotional problems

^8^perceived mental health

### Psychometric properties of the instrument

The initial questionnaire was evaluated through face validity, content validity, exploratory factor analysis with varimax rotation, convergence validity and known groups validity. On the other hand, the reliability was evaluated through internal consistency and stability of the instrument via test-retests. The details of implementation of the psychometric properties are as follows:

#### Face validity

In interviews, 10 women with GDM were asked first to evaluate the questions regarding their level of difficulty, irrelevancy, and ambiguity (qualitative face validity) [20]. Next, they were asked to specify the importance of the items in 5-point Likert scale (quantitative face validity). Using the impact score = importance ∗ frequency and the cut-off point of 1.5, the importance of the items was calculated and evaluated [32].

#### Content validity

The content validity was individually assessed by 10 specialists. A total of 10 specialists in reproductive health, midwifery, and nursing were asked to evaluate the questionnaire in terms of grammar, wording, item allocation (qualitative content validity). Also, the assessment was based on the Waltz & Bausell content validity index (CVI) [33]; the experts scored the relevancy, clarity and simplicity of each item through a four-point Likert scale, and the CVI for each item was calculated by dividing the number of experts who scored items a 3 or 4 by the total number of experts. The statement was accepted if the CVI was ≥0.79 [33]; the necessity of the items was assessed through a three-point rating scale: (i) not necessary, (ii) useful, but not essential (iii) essential. Following the experts’ assessments, a content validity ratio (CVR), for the total scale was computed. According to Lawshe, an acceptable CVR value for 10 experts was 0.62 [33]. Scale-level content validity index (S-CVI) and scale-level content validity ratio (S-CVR) was computed by calculating the mean of CVI and CVR values, where S-CVI > 0.9% is considered acceptable [34].

#### Exploratory factor analysis

exploratory factor analysis was performed to evaluate the validity of the instrument structure. In factor analysis,100–200 samples have been considered to be adequate [35]. Accordingly, 165 pregnant women with GDM were asked to complete the questionnaire. Multi-stage random sampling method was used for selecting mothers. Then, clinics from first, Eastern, Western, Northern and Southern regions of Tehran, under the coverage of Shahid Beheshti University, were classified into 4 categories. Next, from each category, a number of clinics (cluster) were randomly selected, after which inside each cluster the sample was selected based on purposive sampling in proportion to the cluster weight. After the explanation of the study objectives and obtaining informed consent, mothers with GDM completed the questionnaire if they were willing to. Kaiser-Meyer Olkin test was used to assess the sample adequacy. Eigenvalue> = 1 and screen plot were used to determine the factors extracted. The cut-off point was considered 0.4 for the minimum factor load required to maintain the item in the factor.

#### Convergence validity

To assess the convergence validity, diabetes clients’ quality of life questionnaire (DCQOL) was employed. After explaining the study objectives and obtaining informed consent, 85 mothers with GDM willingly completed GDMQ-36 and DCQOL questionnaires. The DCQOL questionnaire is an Iranian instrument whose validity and reliability have been validated and has five subscales including physical, mental, social, economic, and disease and treatment [18]. Then, Pearson correlation coefficient was used to examine the relationship between the five domains of GDMQ-36 and DCQOL questionnaires as well as the total scores of the two questionnaires using SPSS-21. In this study, the correlation coefficients below 0.4, 0.4–0.7, and above 0.7 were considered as low, moderate, and strong, respectively [34].

#### Reliability

Cronbach’s coefficient alpha was used to examine the internal consistency of the subscales and the entire instrument. Values ​​above 0.79 are considered acceptable in descriptive studies [36]. Instrument stability was assessed by the test-retest method and through the completion of the questionnaires by 15 women with GDM within a two-week interval. Intra-class correlation (ICC) was utilized to assess the reliability. If the intra-class correlation is above 0.7, the stability is considered desirable [37].

#### Statistical analysis

Data analysis was performed using SPSS-21. Data analysis was conducted through exploratory factor analysis by principal component method with varimax rotation. Normal distribution of the data was verified using Kolmogorov-Smirnov test. Cronbach’s coefficient alpha and ICC were also calculated. *P* value< 0.05 was considered significant.

**Fig. 1:**
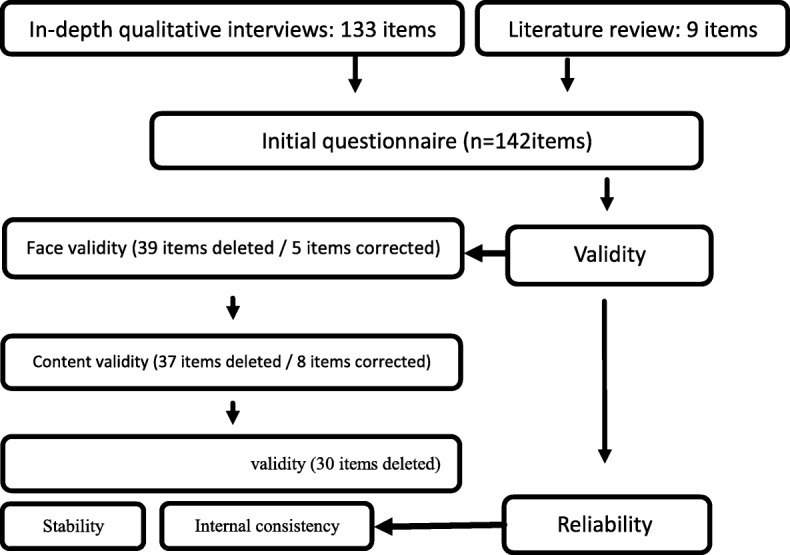
The process of designing and psychometric analysis of the questionnaire

Figure 1 displays the development and psychometric properties of quality of life questionnaire for women with GDM.

#### Ethical considerations

Proposal of the present study was approved by the Vice-Chancellor of the Faculty of Nursing and Midwifery of Shahid Beheshti University of Medical Sciences. The research project was approved by the code of IR.SBMU.PHNM.1395.494 at the Ethics Committee of Shahid Beheshti University of Medical Sciences. Prior to the beginning of the study, the consent of the relevant authorities was obtained. At the beginning of the interviews, purpose of the research, interview method, data confidentiality, and the freedom of choice to enter or quit the study were explained to the participants and their informed consent was obtained.

## Results

### Development of a primary questionnaire

The findings of qualitative study indicated that the quality of life of mothers with GDM is a multidimensional concept. GDM affects different aspects of health and life of mothers suffering from it and leads to physical and psychological problems. Mothers with GDM perceive some limitations like social isolation and playing their maternal and spousal roles or some restrictions as a result of their treatment procedures. In addition, these mothers perceive their pregnancy as high risk because are worried about the outcomes of this disease and concerns about treatment and healthcare service. Using some procedures like adaptation, support and self-care to confront such restrictions and concerns result in coping with this disease. Content analysis led to the extraction of 4 themes including: 1- complications of gestational diabetes (physical and psychological disorders), 2-perceived constraints (social isolation, role playing, and inflexible pregnancy due to treatments), 3-concerns about high risk pregnancy (concern for outcome Disease and treatment) and 4-coping strategies (adjustment, protection and self-care).

Based on the findings of the qualitative phase and literature review, primary question pool of the questionnaire was extracted including 133 items from findings of the qualitative phase of the study and 9 items from the literature review (Table 2). Accordingly, the primary questionnaire was prepared with 142 items. The distribution of 142 items in the questionnaire was as follows: complications of GDM (15 items), perceived constraints (29 items), concerns about high-risk pregnancy (32 items), and coping strategies (66 items). Each item was scored based on5-point Likert scale (strongly agree to strongly disagree) or (always to never).

**Table 2:** Extracted items from the review of the literature to design a questionnaire on “quality of life of mothers with gestational diabetes mellitus”

I prefer to eat something I shouldn’t, rather than tell someone that I have diabetes [26]	
I’m worried about whether I will miss work [26]	
I feel diabetes limits my career [26]	
I feel satisfied with my blood glucose control [28]	
Recording your blood glucose levels in your chart/diabetes diary when your health care personnel asks you to [30]	
I adjust insulin dose based on my blood glucose [30]	
My diet is repetitious and not diversified [38]	
Gestational diabetes has caused me not to enjoy my pregnancy [39]	
I feel that my pregnancy is an abnormal pregnancy [40]	

#### Face validity

Demographic information on patients participated in the face validity are shown in Table 3. In qualitative face validity, according to the participants’ comments (10 pregnant women with GDM), a number of items were corrected. In the quantitative face validity, on the other hand, 39 items were eliminated with a cut-off point of 1.5 as an acceptable impact score to maintain the item (reducing 142 to 103 items).

**Table 3:** Demographic characteristics of women with GDM that participated in psychometric testing steps

Characteristics	Face validity (*n* = 10)	Factor analysis And Known group validity (*n* = 165)	Convergence validity (*n* = 85)	Internal consistency (*n* = 30)	Stability (*n* = 15)
Age (years)					
Mean (SD)	29.6 (4.69)	31.79 (5.30)	29.87 (5.33)	28.20 (5.88)	28.40 (5.91)
Range	23–38	19–44	19–40	19–39	20–38
Gestational age (weeks)					
Mean (SD)	30.80 (3.76)	31.00 (3.52)	30.40 (3.74)	31.33 (3.72)	31.2 (3.70)
Range	27–36	26–39	26–39	26–37	26–36
Level of education					
Elementary	6 (60%)	67 (40.6%)	21 (24.7%)	12 (40%)	7 (46%)
High school	2 (20%)	60 (36.4%)	36 (42.4%)	10 (33.3%)	5 (33%)
College/university	2 (20%)	31 (18.8%)	23 (27.1%)	8 (26.7%)	3 (20%)
postgraduate	0 (0%)	7 (4.2%)	5 (5.9%)	0 (0%)	0 (0%)
Employment status					
Employment	8 (80%)	18 (10.9%)	12 (14.1%)	4 (13.3%)	2 (13.3%)
Housekeeper	2 (20%)	147 (89.1%)	73 (85.9%)	26 (86.7%)	13 (86.7%)
Economic status					
Poor	4 (40%)	64 (38.8%)	23 (27.1%)	12 (40%)	6 (40%)
Moderate	5 (50%)	79 (47.9%)	48 (56.5%)	15 (50%)	9 (60%)
good	1 (10%)	22 (13.3%)	14 (16.5%)	3 (10%)	0 (0%)
Gravida					
nuliparus	4 (40%)	49 (29.7%)	37 (43.5%)	10 (33.3%)	2 (13.3%)
multiparus	6 (60%)	116 (70.3%)	48 (56.5%)	20 (66.6%)	13 (86.7%)
Hospitalization					
Yes	3 (30%)	63 (38.2%)	28 (32.9%)	11 (36.7%)	4 (26.7%)
no	7 (70%)	102 (61.8%)	57 (67.1%)	19 (63.3%)	11 (73.3%)
Treatment					
Diet	7 (70%)	79 (47.9%)	47 (55.3%)	17 (56.7%)	11 (73.3%)
Medical treatment	3 (30%)	86 (52.1%)	38 (44.7%)	13 (43.3%)	4 (26.7%)

#### Content validation

In the qualitative content validity, according to the suggestions of experts (10 specialist), some items were revised, at which phase 5 items related to costs were summed up in 1 item, whereby the number of the items reduced from 103 to 99. In the quantitative content analysis, the Content Validity Ratio (CVR) and Content Validity Index (CVI) were calculated for each item, and considering the appropriate cut-off point, the number of items was curtailed from 103 to 66, with S-CVI and S-CVR obtained as 0.99and 0.73, respectively.

#### Factor analysis

For exploratory factor analysis, 165 women with GDM completed a 66-item questionnaire. Demographic information on patients participated in the factor analysis are shown in Table 3. The recommended number of respondents for factor analysis is between 100 and 200 subjects [41]. The value of 0.80 obtained from KMO test and the significance of the Bartlett test results (*p* < 0.001) suggested that the sampling was adequate. In the exploratory factor analysis, considering 0.4 as the minimum factor load, 30 items were deleted, with 36 items left. Decision on the number of factors for rotation was based on the special value of above 1, with 10 factors accounting for 61.71% of the observed variance; which seems high considering the theoretical basis. Accordingly, five factors were considered as defaults in factor analysis to simplify and make the constructive factors of the instrument developed interpretable, and given the low explaining power of the last factors, taking into account the consistency of the extracted factors with the concept and dimensions of the quality of life of mothers with GDM.

Factor analysis with 5 constant factors was repeated with varimax rotation. Based on the 5 constant factors, 46.68% of variance was explained. The highest variance was assigned to the factor 1 (concerns related to high risk pregnancy), while the minimum variance was related to factor 5 (support) (Table 4).

**Table 4:** Initial eigenvalues for the Quality Of Life Questionnaire for women with Gestational Diabetes Mellitus

Component	Initial Eigenvalues			Extraction Sums of Squared Loadings			Rotation Sums of Squared Loadings		
	Total	% of Variance	% Cumulative	Total	% of Variance	% Comulative	Total	% of Variance	% Comulative
1	8.49	22.96	22.96	8.49	22.96	22.96	5.51	14.90	14.90
2	2.57	6.95	29.91	2.57	6.95	29.91	3.62	9.78	24.69
3	2.49	6.74	36.65	2.49	6.74	36.65	2.86	7.73	32.42
4	2.03	5.49	42.14	2.03	5.49	42.14	2.68	7.26	39.68
5	1.67	4.53	46.68	1.67	4.53	46.68	2.58	6.99	46.68

Based on factor analysis, 5 factors were extracted for the quality of life questionnaire for women with GDM (Table 5). As shown in Table 5, some of the items with different factor loads are placed across several factors. Although it was attempted to place the items in factors with the largest factor load, the item “I’m concerned about delayed wound healing” was passed to a factor with a lower factor load since it was not consistent the factor with the highest factor load. The items that were not loaded on any of the factors were excluded from the questionnaire whereby the questionnaire was reduced to 36 questions. Subsequently, considering the research team’s commentary, each factor was named according to its items. The 5 factors in quality of life questionnaire for women with GDM included concerns about high-risk pregnancy (11 items), perceived constraints (8 items), GDM complications (6 items), medication and treatment (5 items), and support (6 items).

**Table 5:** Matrix of Rotated Factors with Varimax Rotation and Factor Load rates of Items in Each Factor for the Quality Of Life Questionnaire for women with Gestational Diabetes Mellitus

Raw	Item	Factor load				
		1	2	3	4	5
1	I’m concerned about premature birth of my baby	0.846				
2	I’m concerned about the loss of fetal movement	0.795				
3	I’m concerned about the fetal death	0.775				
4	I’m concerned about fetal abnormalities	0.765				
5	I’m concerned about my baby’s blood sugar drop after birth	0.655				
6	I have to visit doctors with different specialties	0.601				
7	I’m concerned about poor weight gain	0.572				
8	I’m concerned that diabetes would be transmitted to my baby	0.558				
9	I have to visit the doctor frequently	0.554				
10	I’m concerned about fetal and baby weight gain	0.524				
11	I’m concerned about delayed wound healing	0.436			0.439	
12	My diet is repetitious and not diversified		0.721			
13	I faced constraints on my favorite foods and fruits		0.705			
14	My food is separated from the family meal		0.612			
15	The family basket has changed due to my diet		0.524			
16	To measure fasting blood sugar, I should fast for a long time and endure hunger		0.518			
17	My sexual activity has decreased due to GDM		0.480			
18	I use fruits and food with low and determined amounts		0.457			
19	I go less often to the party and restaurants due to GDM		0.401			
20	I have feelings of thirst and dry mouth			0.731		
21	I repeatedly go to the bathroom			0.695		
22	I have blood sugar drop			0.628		
23	I get angry soon			0.580		
24	I feel depressed			0.579		
25	My mind is obsessed with the disease		0.414	0.465		
26	Insulin injection is difficult for me at specific times of the day				0.760	
27	Insulin injections for several times is difficult and time consuming for me				0.755	
28	Frequent blood glucose test is difficult for me				0.510	
29	I’m concerned about drug side effects on my fetus				0.508	
30	I adjust insulin dose based on my blood glucose				0.499	
31	People’s empathy helps me to tolerate the disease					0.678
32	My spouse mental and emotional support helps me tolerate the disease easier					0.671
33	The information from healthcare workers about the disease has helped me					0.605
34	The positive experience of the people around me about the disease has helped me					0.594
35	The information I receive about the disease from the media and the Internet has helped me					0.560
36	Prayer with God has helped me tolerate the disease					0.431

#### Convergence validity

To assess the convergence validity, the quality of life questionnaire for women with GDM and Diabetes Clients Quality Of Life questionnaire were completed by 85 pregnant women with GDM. Demographic information on patients participated in the convergence validity are shown in Table 3. The correlation between the scores of the different domains of the two questionnaires and the correlation of the two questionnaires were examined via Pearson test (Table 6). The results showed a moderate and significant relationship between the total score of the two questionnaires (r = 0.64).

**Table 6:** Correlation Coefficient between the GDMQ-36 factors and the DCQOL subscales (*n* = 85)

Subscales of questionnaire			Diabetes Clients Quality Of Life questionnaire (DCQOL)			
		Physical	Psychological	Social	Economic	Disease and treatment
Gestational Diabetes Mellitus Quality Of Life-36 Item (GDMQ-36)	concerns about high-risk pregnancy	0.04	0.14	−0.04	0.06	0.39**
	perceived constraints	−0.02	0.10	−0.18	−0.006	0.67**
	GDM complications	0.14	0.65**	0.11	0.21*	−0.13**
	medication and treatment	0.10	−0.01	0.005	−0.05	0.46**
	support	0.06	0.005	− 0.43**	−0.06	0.04

*Significant at the *p* < 0.05

**Significant at the *p* < 0.01

#### Reliability

Demographic information on patients participated in the reliability are shown in Table 3. The internal consistency of the questionnaire was evaluated using the Cronbach’s coefficient alpha. The coefficient of 0.93 for the entire instrument and coefficients of 0.77 to 0.90 for the sub-scales showed proper internal consistency. Tool stability was tested by a test-retest method via intra-class correlation coefficient. ICC of 0.95 for the entire instrument and coefficients of 0.85 to 0.98 for sub-scales revealed acceptable stability of the questionnaire (Table 7).

**Table 7:** Reliability measures for the GDMQ-36

subscales	Cronbach’s alpha	intra-class correlation coefficient (ICC)
concerns about high-risk pregnancy	0.90	0.98
perceived constraints	0.80	0.95
GDM complications	0.85	0.90
medication and treatment	0.77	0.85
support	0.84	0.92
Total	0.93	0.95

After evaluation of the validity and reliability, the final questionnaire quality of life on GDM (GDMQ-36) was developed with 36 items and 5 factors including “concerns about high-risk pregnancy”, “perceived restrictions”, “disease complications”, “medication and treatment” and “support” (Table 8).

**Table 8:** Final questionnaire quality of life on GDM (GDMQ-36)

After Gestational Diabetes Mellitus		strongly disagree	disagree	have no idea	agree	strongly agree
1	I’m concerned about premature birth of my baby					
2	I’m concerned about the loss of fetal movement					
3	I’m concerned about the fetal death					
4	I’m concerned about fetal abnormalities					
5	I’m concerned about my baby’s blood sugar drop after birth					
6	I have to visit doctors with different specialties					
7	I’m concerned about poor weight gain					
8	I’m concerned that diabetes would be transmitted to my baby					
9	I have to visit the doctor frequently					
10	I’m concerned about fetal and baby weight gain					
11	I’m concerned about delayed wound healing					
12	My diet is repetitious and not diversified					
13	I faced constraints on my favorite foods and fruits					
14	My food is separated from the family meal					
15	The family basket has changed due to my diet					
16	To measure fasting blood sugar, I should fast for a long time and endure hunger					
17	My sexual activity has decreased due to GDM					
18	I use fruits and food with low and determined amounts					
19	I go less often to the party and restaurants due to GDM					
20	I have feelings of thirst and dry mouth					
21	I repeatedly go to the bathroom					
22	I have blood sugar drop					
23	I get angry soon					
24	I feel depressed					
25	My mind is obsessed with the disease					
26	Insulin injection is difficult for me at specific times of the day					
27	Insulin injections for several times is difficult and time consuming for me					
28	Frequent blood glucose test is difficult for me					
29	I’m concerned about drug side effects on my fetus					
30	I adjust insulin dose based on my blood glucose					
31	People’s empathy helps me to tolerate the disease					
32	My spouse mental and emotional support helps me tolerate the disease easier					
33	The information from healthcare workers about the disease has helped me					
34	The positive experience of the people around me about the disease has helped me					
35	The information I receive about the disease from the media and the Internet has helped me					
36	Prayer with God has helped me tolerate the disease					

### Scoring procedure

The final questionnaire for quality of life of mothers with GDM consists of 36 questions in 5 domains, based on 5-point Likert scale (strongly agree to strongly disagree) with a score range of 1 to 5. This procedure is used for the items in the domains of concerns about high-risk pregnancy, perceived constraints, complications of GDM, and medication and treatment, with the exception of item 29, “I adjust insulin dose based on my blood glucose”(strongly agree = 1, strongly disagree = 5). In the domain of support, the answer “strongly agree” scores 5 while the answer “strongly disagree” scores 1. Mothers who do not receive insulin,scores of these questions is considered 3(have no idea) .

The total score of the questionnaire, based on the above explanations is 36–180, with higher scores indicating greater quality of life. Due to the diversity of the domains studied and the response scales chosen for each domain, a standardization method of 0 to 100 was used for better understanding the scoring procedure and comparison of the questionnaire’s different sub-scale scores. To convert the scores of the sub-scales and the entire questionnaire to a score of 0 to 100, the following conversion formula was used.

Adjusted score = (acquired raw score/maximum possible score)*100.

Using this formula, a score of 0 to 100 is obtained in each of the sub-scales. Higher scores represent a better status in the subscale. The total score of the instrument is computed by calculating the average of the total modified scores of the instrument. Higher scores in the entire instrument represent better quality of life. Score range of questionnaire for quality of life of mothers with GDM (GDMQ-36) are shown in Table 9.

**Table 9:** Score range of questionnaire for quality of life of mothers with GDM (GDMQ-36)

Domains	Score range	Adjusted score
Concerns about high-risk pregnancy	11–55	(acquired raw score/maximum possible score)*100
Perceived constraints	8–40	
Complications of GDM	8–30	
Medication and treatment	5–25	
Support	6–30	
Total	36–180	

## Discussion

In this study, using a qualitative and quantitative approach, a valid, reliable, and practical questionnaire was developed for assessing the quality of life of women with GDM (GDMQ-36). In this questionnaire, the quality of life of mothers with GDM was influenced by concerns about high-risk pregnancy (11 items), perceived constraints (8 items), complications of GDM (6 items), medication and treatment (5 items), and support (6 items).

One of the most important factors in GDMQ-36 questionnaire is high-risk pregnancy related concerns, which claimed the highest percentage of variance among the tool’s factors. Most of the items in this factor are concerns about the fetal health or baby health, and the most frequent factor load is the concern about the premature birth and reduced fetal movements. In various studies, concerns about neonatal health before and after birth such as preterm labor or constraints on fetal development and mortality have been raised and most of the mothers are mainly concerned about the fetal and neonatal health [42–44].

Another factor of the GDMQ-36 questionnaire was perceived constraints. The most frequent factor load in this factor was dietary constraints including limited diet diversity and constraints on favorite fruits and foods. In the qualitative study, most participants found it confusing and frustrating that they could only eat a very small portion at once, or could not eat particular fruits at all which they enjoyed prior to being diagnosed with GDM [38]. These constraints are specific to mothers with GDM who have to follow a special diet and the treatment to maintain their own and the fetal health. Such constraints can affect the quality of life of mothers with GDM. Among other constraints in GDMQ-36 questionnaire are sexual constraints which can affect the quality of life of mothers with GDM. Hyperglycemia can result in elevated serum prolactin levels and lead to neurotransmitter changes, which are potentially related to sexual dysfunction. Other causes of sexual dysfunction in these women may be the stress and anxiety sufferance because of GDM diagnosis [45].

Another factor in GDMQ-36 questionnaire is the disease complications including physical and psychological complications. Complications of gestational diabetes are the negative changes in various aspects of the person’s health caused by the disease, which can reduce the mothers’ quality of life. Due to the multidimensionality of quality of life, poor physical and mental health can affect the quality of life. The most frequent factor load in this factor was related to physical complications such as thirst and dry mouth and frequent urination. In a study examining the clinical and laboratory symptoms of glucose tolerance, over-hydration and frequent urination were the most important findings [46].

Another factor in GDMQ-36 questionnaire is medication and treatment. When the blood glucose cannot be controlled by diet, mothers have to administer insulin injections. The most frequent factor load in this structure was the problem of insulin injections. Repeated and timely injections of insulin, and frequent blood glucose tests in the laboratory or by a glucose meter require time management and can affect the mothers’ quality of life. In a study, most participants felt that they were controlled by the diabetes and they are entrapped in a compulsory lifestyle. This compulsory life-style involved continuous monitoring of blood glucose, insulin infusion, and insulin dose adjustment [47]. This change in the lifestyle that is unfamiliar and sometimes unwanted can affect the quality of life of mothers with GDM. This has been considered in the domain of medication and treatment of GDMQ-36 questionnaire.

The last factor in GDMQ-36 questionnaire is support. The most frequent factor load in this factor is the empathy of the intimates and full support of the spouse. The support of friends and family members for mothers with GDM is effective in choosing a healthy lifestyle [48]. In the study of Han et al., the first requirement for mothers with GDM to face the disease problems was the family or spouse support [49]. Therefore, the domain of support in GDMQ-36 questionnaire may improve the quality of life of mothers with GDM.

Evaluating and recording health-centered quality of life based on quality of life assessment questionnaires has been of great importance. In previous studies, only general quality of life assessment questionnaires such as SF-36 and WHO-QOL-Bref have been used to assess the quality of life of mothers with GDM [10–12]. Factor Comparison of GDM-36 questionnaire with general questionnaires suggested that GDM-36 factors are similar to some aspects of WHOQOL-Bref [25] and SF-36 [24], especially the ​​mental and social health aspects. Although some of the items in GDMQ-36 questionnaire are consistent with the general questionnaire, the nature of the items in the general questionnaire is different from that of the questionnaire developed in this study. Specifically, the items in GDMQ-36 questionnaire are all in the context of GDM, but the items in the general questionnaires are designed in a way to be applicable to all communities. Public questionnaires (such as SF-36) are designed with no regard to the unique experiences of pregnancy. Although there are studies that show the adequacy of these questionnaires for different communities, their adequacy for the pregnant population has not been determined [13]. In their study, Otchet et al. stated that the SF-36 health questionnaire of pregnant women has some constraints [50].

Other specific questionnaires, in this regard, include the pregnant women’s quality of life and diabetes’ quality of life questionnaire.

The pregnant women’s quality of life questionnaire (QOL-GRAV) is an instrument with 9 questions originally developed in Czech Republic. According to its designers, it is a supplementary tool for the general quality of life questionnaire of World Health Organization (WHO) [15]. Comparison of this tool with GDM-36 questionnaire revealed that QOL-GRAV could not be potentially suitable for the quality of life assessment of mothers with GDM as it ignores many concerns and constraints which can affect the quality of life of mothers with GDM. In thin questionnaire, there is only one question, i.e. “How concerned are you about carrying a successful pregnancy?” that can be partly consistent with the domain “Concerns about high-risk pregnancy” in GDM-36 questionnaire. Another questionnaire on pregnant women’s quality of life has been developed in Iran which includes 65 questions in three domains of physical, psychological, and social problems; concern and worries; and adaptation [14]. During pregnancy, physiologically due to hormonal changes, some physical or psychological complications occur in pregnant mothers. These issues have been addressed in this questionnaire, but the complications assessed in GDM-36 are the result of the increased or decreased blood glucose levels. The domain of concerns and worries in the pregnant women’s quality of life questionnaire, in some cases, were similar to concerns about high-risk pregnancy in GDM-36 questionnaire. However, in the pregnant women’s quality of life questionnaire, most concerns were about childbirth than the fetal health, as pregnant mothers’ quality of life questionnaire is only suitable for mothers who have a healthy and safe pregnancy.

Audit of Diabetes-Dependent Quality Of Life (ADDQOL) is a 13-item questionnaire developed by Bradley et al. [16]. Also, Diabetes Quality of Life (DQOL) questionnaire has 16 questions developed by Lee et al. [17].Since diabetes is a chronic disease and can have a major impact on the social dimension and long-term consequences on the life of the patients, the questionnaire questions are more about social problems or long-term concerns of the disease. Therefore, these questionnaires may not be able to assess the pregnant mothers’ quality of life, especially those diagnosed with diabetes during the pregnancy.

The face, content, exploratory factor analysis, and reliability are required to assess psychometric properties of a questionnaire [20]. In this study, qualitative and quantitative face validity, qualitative and quantitative content validity, exploratory factor analysis, convergence validity, and reliability were evaluated for GDMQ-36 questionnaire. For its face validity, after correcting the ambiguous items, the impact factor was calculated for each item, where items with impact factor of less than 1.5 were omitted. In content validity, CVR and CVI were calculated for each item, where items with CVR of less than 0.62 and CVI of less than 0.79 were removed, after consultation with the research team. The review of the items deleted in face and content validity evaluation phase indicated that their concepts were explained by the remaining variables. S-CVI was equal to 0.99 demonstrating that the entire tool has a desirable and robust strong validity [34].

Exploratory factor analysis was used for validity of GDMQ-36 questionnaire. Factor analysis is performed in two general forms called exploratory and confirmatory factor analysis. The confirmatory factor analysis is used to confirm previous work. In exploratory factor analysis, the researcher seeks to discover the underlying structure of a relatively large set of variables, with the initial assumption being that each variable may be related to each of the factors. In other words, the researcher has no initial theory in this method [34]. In exploratory factor analysis of GDMQ-36, five factors explained 46.68% of the total variance of the questionnaire through exploratory factor analysis.

Diabetes clients’ quality of life (DCQOL) questionnaire was used for convergent validity of GDMQ-36 questionnaire. Since there was no quality of life questionnaire for women with GDM, diabetes’ quality of life questionnaire was used. Among the diabetes’ quality of life questionnaires, Diabetes clients’ quality of life (DCQOL) questionnaire proved to be more appropriate, since this questionnaire was developed and assessed regarding its psychometric properties in Iran [18] and has been closer to GDMQ-36 questionnaire in terms of culture. Statistical analysis showed a moderate and significant relationship between the two questionnaires which was acceptable [51]. Factors of concerns about high-risk pregnancy, perceived constraints, as well as medication and treatment in GDMQ-36 questionnaire had a significant relationship with factors of disease and treatment in the DCQOL questionnaire. Comparison of the items of these factors revealed that the concept of some items of the disease and the treatment factor in the DCQOL questionnaire existed in the three domains of GDMQ-36 questionnaire. However, the items of DCQOL questionnaire captured more general concerns and did not take into account pregnancy concerns. The disease complications of GDMQ-36 questionnaire had a significant correlation with the psychological domain of the DCQOL questionnaire, while the physical complications of the DCQOL questionnaire included those more related to the physical problems of chronic diseases. The support factor of our questionnaire had a significant and inverse relationship with the social domain of the DCQOL questionnaire. Specifically, in our questionnaire, the items about support were positive while in the DCQOL questionnaire, the items associated with support were negative, and it was of the long-term social complications of the disease.

One strong point of this study was the use of exploratory mixed methods including qualitative and quantitative phases for development and assessment of psychometric properties of the questionnaire. The participants of this study were mothers suffering GDM with the highest diversity in age, education, economic status, gestational age, and type of treatment (diet or insulin, inpatient or outpatient treatment).This makes it easier to evaluate the quality of life of mothers with GDM through GDMQ-36 questionnaire. Nevertheless, this study had also some limitations. The most important was that the questionnaire was developed in Iran, thus requiring translation and revalidation in other communities. It seems that a well-designed questionnaire can be used in various communities after being fully translated and evaluated by the professionals in those communities [52].

## Conclusion

GDMQ-36 is a simple, valid, and reliable tool for assessing the quality of life of women with GDM. GDMQ-36 may help healthcare providers and health policy makers to identify and assess the different dimensions of quality of life of mothers with GDM and to adapt their measures to improve the health and quality of life of these mothers. Different screening and treatment methods have been proposed for GDM. Policymakers can use this questionnaire to compare different types of GDM screening methods or different therapeutic approaches (diet, drug administration, oral medications), and use a method for screening and treatment with the minimum effect on these mothers’ quality of life.

## Data Availability

The datasets used and/or analysed during the current study are available from the corresponding author on reasonable request.

## Abbreviations

ADDQOL: Audit of Diabetes-Dependent Quality Of Life
CVI: Content Validity Index
CVR: Content Validity Ratio
DCQOL: Diabetic Clients Quality Of Life
DQOL: Diabetes Quality of Life
GDM: Gestational Diabetes Mellitus
GDMQ-36: Gestationla Diabetes Mellitus Quality of life questionnair 36 item
ICC: Intra-Class Correlation
OGTT: Oral Glucose Tolerance Test
QALY: Quality Adjusted Life Years
QOL-GRAV: Pregnant women’s quality of life questionnaire
S-CVI: Scale-level Content Validity Index
S-CVR: Scale-level Content Validity Ratio
SF-36: 36-Item Short-Form Health Survey
WHO-QOL-Bref: qWorld Health Organization quality of life
